# A biomechanical approach to understand the ecomorphological relationship between primate mandibles and diet

**DOI:** 10.1038/s41598-017-08161-0

**Published:** 2017-08-21

**Authors:** Jordi Marcé-Nogué, Thomas A. Püschel, Thomas M. Kaiser

**Affiliations:** 1 Centrum für Naturkunde, University of Hamburg, Martin-Luter-King-Platz, 3 20146 Hamburg, Germany; 2 School of Earth and Environmental Sciences, University of Manchester, Oxford Road, M13 9PL Manchester, UK

## Abstract

The relationship between primate mandibular form and diet has been previously analysed by applying a wide array of techniques and approaches. Nonetheless, most of these studies compared few species and/or infrequently aimed to elucidate function based on an explicit biomechanical framework. In this study, we generated and analysed 31 Finite Element planar models of different primate jaws under different loading scenarios (incisive, canine, premolar and molar bites) to test the hypothesis that there are significant differences in mandibular biomechanical performance due to food categories and/or food hardness. The obtained stress values show that in primates, hard food eaters have stiffer mandibles when compared to those that rely on softer diets. In addition, we find that folivores species have the weakest jaws, whilst omnivores have the strongest mandibles within the order Primates. These results are highly relevant because they show that there is a strong association between mandibular biomechanical performance, mandibular form, food hardness and diet categories and that these associations can be studied using biomechanical techniques rather than focusing solely on morphology.

## Introduction

Diet is regarded as one of the main factor underlying the behavioural and ecological differences among living primates, and consequently primate diets have been more exhaustively documented than any other aspect of their behaviour^1^. A substantial proportion of physiological and anatomical adaptations have as their fundamental objective the transformation of the ingesta that animals consume. Most primates have been habitually interpreted as mainly adapted to fruit consumption^2^, however it has been also acknowledged that some species occupy specific dietary niches ranging from omnivory to the pure folivory^1^. Consequently, primates have been classified into three main diet categories: frugivores, folivores, and omnivores. These broad categories are coherent with much of the structural and nutritional characteristics of the food items observed in primates, and thus frugivores, folivores, and omnivores have characteristic features that enable them to process their different diets. Furthermore, some primates are adapted to the consumption of hard items (durophagy; hard-food eaters) whereas others are classified as soft-food consumers^3^.

The relationship between primate mandibular form and loading during biting has been analysed by numerous studies^4^. This interest regarding shape and function in the mandible has not been restricted to primates; in fact, other mammalian clades such as Artiodactyla^5, 6^, Chiroptera^7^, and Carnivora^8, 9^ have been studied as well. The close interaction between the mammalian feeding mechanism and the ingesta it processes represents a unique opportunity to study ecomorphological adaptations in extant species and potentially acquire valuable tools for the reconstruction of feeding behaviours in extinct taxa as well.

The main function of the mammalian mandible is to transfer the forces generated by the masticatory muscles to the ingesta via the teeth. It has been proposed that mandibular shape is mostly involved in ensuring that the forces are transmitted without being dissipated or causing the mandible to fail structurally^10^. Mandibular shape is related to diet through the frequency and magnitude of adductor muscle forces engaged during various oral activities. The greater the forces required to fracture food items (or their protective structures), and the more repeatedly such forces need to be produced (e.g. through repetitive biting), the stronger the mandible has to be to maintain its structural integrity^11^. This has been experimentally tested by feeding animal with diets of different hardness. Studies on primates and marsupials have demonstrated that durophagy elicits greater cortical bone in the mandibular body when compared to control subjects fed with a softer diet^12^. In consequence, species that regularly consume hard items are expected to exhibit jaws that are better able to resist these mechanical loadings.

This relationship between diet and morphology has been studied using a wide array of techniques and approaches. These include comparative functional morphology and biomechanics (e.g. refs 13 and 14) dental wear and tear (e.g. refs 15 and 16) and dental morphology and allometry^17, 18^. Finite Element Analysis (FEA) has also been applied in a wide diversity of vertebrates providing new insights to explore the function, morphological evolution, and adaptations of different bony structures^19^. Particularly in primate mandibles, it has been used to analyse the biomechanics during chewing because the jaw may be more susceptible to failure than the cranium^20^. Many studies have focused on the mandible rather than the skull because this latter structure shows a morphology associated with multiple functions, while the jaw is mostly involved in food consumption, and hence it would be expected that its morphology better reflects diet adaptations^21^. Early works were carried out in primates to study mandibular biomechanics in extant and extinct taxa during chewing using formulations from classic mechanics^22–25^. More recently, some studies have been performed focusing on the biomechanics of the mandible in different families of mammals using FEA to study the mandible during chewing in 3D models^26^, as well as in plane models^27–29^. For instance, Wroe *et al*. studied the biomechanics of the jaw and skull in *Homo sapiens* compared to other primates using FE models where the stress patterns obtained in each species were interpreted as a sign of relative strength^20^. Nevertheless, most of the works analysing primates using FEA have focused on the biomechanical performance of the skull^30–34^.

Previous studies have analysed primate mandibular biomechanics^4^, however most of these studies compared few species within a limited phylogenetic extent. When considering that the biomechanical aspects of the mandible are crucial to understand dietary adaptation and evolution in primates^20, 22^ it is logical to focus this study on generating several FEA models representing primate jaw diversity. These models were used to test the hypothesis that hard food-eaters have a stiffer jaw as compared to those that eat softer diets, while also testing the expectation that folivores should exhibit weaker mandibles when compared to omnivores due to the occasional consumption of animal tissues by the latter group.

## Materials and Methods

Finite Element Analysis (FEA) enable the observation of stress distribution patterns in the analysed specimens by simulating loadings and forces involved in mastication. In this work, plane models of the mandible individuals belonging to different primate species were analysed by obtaining the von Mises stress patterns. Plane elasticity^35^ has been widely used in palaeontology and biology as a proxy of the biomechanical performance of mandibles^27–29^, although there are no previous studies applying these methodologies in primates. In the present study, a plane stress analysis was carried out, in which the analysed structural elements have one dimension (i.e. thickness) smaller than the other two, thus the stresses are negligible with respect to the smaller dimension. In this case, the thickness of each mandible was assumed to be constant and based in the mean value of three measurements in different points of the jaws (Table S1 for the THK1, THK2 and THK3 values).

A total number of 31 extant primate species were analysed. All specimens examined (Table 1) are housed either at the Museum für Naturkunde in Berlin, Germany or the Centrum für Naturkunde in Hamburg, Germany.

**Table 1:** List of primate species used in the present study.

SPECIE	Acession number	FAMILY	DIET	HARDNESS
*Alouatta seniculus*	ZMH-S 3495	Atelidae	L	S
*Aotus trivirgatus*	ZMH-S 5276	Cebidae	O	H
*Ateles geoffroyi*	ZMH-S 2994	Atelidae	F	H
*Brachyteles arachnoides*	ZMB-Mam- 36455	Atelidae	F	H
*Brunopithecus hoolock*	ZMH-S 4821	Hylobatidae	F	S
*Callithrix jacchus*	ZMH-S 3299	Cebidae	O	H
*Cebus apella*	ZMH-S 3567	Cebidae	O	H
*Cebus capucinus*	ZMH-S 3950	Cebidae	O	H
*Cercocebus torquatus*	ZMH-S 6381	Cercopithecoidea	O	H
*Chlorocebus aethiops*	ZMH-S 4555	Cercopithecoidea	O	S
*Eulemur fulvus*	ZMB-Mam- 7768	Lemuridae	F	S
*Gorilla gorilla*	ZMH-S 6992	Hominoidae	L	S
*Hapalemur griseus*	ZMB 35263	Lemuridae	L	S
*Homo sapiens*	ZMH-S 9537	Hominoidae	O	S
*Hylobates lar*	ZMH-S 7013	Hylobatidae	F	S
*Hylobates moloch*	ZMH-S 8369	Hylobatidae	F	S
*Hylobates muelleri*	ZMB-Mam- 7863	Hylobatidae	F	S
*Lemur catta*	ZMH-S 3259	Lemuridae	F	S
*Macaca fascicularis*	ZMH-S 10191	Cercopithecoidea	O	S
*Macaca fuscata*	ZMH-S 9495	Cercopithecoidea	L	H
*Macaca mulatta*	ZMH-S 4755	Cercopithecoidea	L	H
*Macaca nemestrina*	ZMH-S 3274	Cercopithecoidea	F	H
*Nycticebus coucang*	ZMH-S 4807	Lorisidae	O	H
*Pan troglodytes*	ZMH-S 2756	Hominoidae	F	S
*Papio cynocephalus*	ZMH-S 6802	Cercopithecoidea	L	H
*Papio ursinus*	ZMB-Mam- 18047	Cercopithecoidea	O	S
*Pithecia pithecia*	ZMH-S 7625	Pitheciidae	F	H
*Pongo pygmaeus*	ZMH-S 9395	Hominoidae	F	H
*Saimiri sciureus*	ZMH-S 7633	Cebidae	O	H
*Therophitecus gelada*	ZMH-S 3273	Cercopithecoidea	L	H
*Trachypithecus cristatus*	ZMH-S 1818	Cercopithecoidea	L	S

Museum Acronyms: ZMH = Centrum für Naturkunde, Hamburg, Germany; ZMB = Museum für Naturkunde Berlin, Germany. Families are according the classification of Wilson and Reader^72^ and Arnold *et al*.^52^. Diet: O = Omnivore; F = Frugivore; L = Folivore. Hardness: H = Hard-food eaters; S = Soft-food eaters.

### Diet classification

Primate species were classified according to their diet based on the available literature into three main groups: folivores, frugivores and omnivores (Table 1). Additionally, the analysed species were also categorized according to the relative toughness of their typical food into the two following categories: hard-food and soft-food eaters (Table 1).

The diet classification was established according to the percentage of different foods in the diet^36^. A species was classified as frugivore when more than 60% of its diet is composed by fruit (*Ateles geffroyi, Eulemur fulvus, Brunopithecus hoolock, Hylobates moloch, Hylobates muelleri, Hylobates lar, Lemur catta, Macaca nemestrina, Pan troglodytes and Pongo pygmaeus*) or alternatively, when there was a well-established consensus in the literature: *Brachyteles. arachnoides* ^37^, *Pithecia monachus* ^38^. A species was categorized as omnivorous when besides of other components the diet comprises more than 30% of vertebrates and/or invertebrates, as well as exhibiting a great variety of ingesta (*Aotus trivirgatus, Callithrix jachus, Cebus apella, Cebus capucinus, Cercocebus torquatus, Chlorocebus aethiopos, Macaca fascicularis, Nycticebus coucang, Papio ursinus and Saimiri sciureus)*. *Homo sapiens* was also classified as an omnivore. Species were classified as folivores when leaves make up more than 40% of their diets (*Gorilla gorilla, Hapalemur griseus, Macaca fuscata, Macaca mulatta, Papio cynocephalus, Trachypithecus crystatus* and *Theropitecus. gelada*). *Alouatta seniculus* was categorised as folivore as well because the quantity of fruits in its diet is lower than 50% and also because it does not consume a significant amount of seeds.

The classification of the relative toughness of the typical food of the different primate species (i.e. hard or soft) was based on the work of Kupczik *et al*.^3^. Most of the species analysed here were present in that study, although there were some specimens that had to be categorised using other sources. *B. arachnoides* was classified according to de Carvalho *et al*.^37^, while all the species that show seed consumption in Wilman *et al*.^36^ were classified as hard eaters. Most of the soft eaters were categorised according to Kupczik *et al*.^3^ as well, whilst *C. aethiops, G. gorilla, P. ursinus and T. crystatus* were classified based in the absence of seeds in their diets, as well as according to mainly the soft foods they consume based on the data provided by Wilman *et al*.^36^.

### Reconstruction of the models

The mandibles of the different primate species were analysed as planar 2D models in FEA using the software ANSYS v.16.1 for Windows 7 (64-bit system). The steps followed to generate the digitals models from pictures were based in the methodology proposed by Fortuny *et al*.^39^ and are described as follows:

1. Specimens were orientated according to the Frankfurt horizontal plane. The skulls and mandibles were positioned in anatomical orientation and central occlusion. Then, photographs were taken from a lateral perspective.
2. The masseter and temporalis muscle insertion areas were identified using Photoshop v.8.0.1 (Adobe Systems). The vector directions of each muscle were estimated using the area centroid of the muscle attachment areas.
3. Smoothed planar surfaces of the muscular areas were generated in Rhinoceros v.4.0 (McNeel & associates) using the information from the previous step.
4. The geometrical properties (i.e. distances, areas) were estimated in Rhinoceros v.4.0 and were also used to place the centroid of the muscular areas.
5. The FEA model generation, as well as the stress distribution results for each mandible were obtained in ANSYS v.16.1.

In order to take the photographs in the most consistent way, some procedures were followed to standardise images following the recommendations proposed by De Esteban-Trivigno^40^.

The FEA models of the various mandibles were meshed using 8-node quadrilateral plane elements (QUAD8), creating the quasi-ideal mesh (QIM) proposed by Marcé-Nogué *et al*.^41^. This particular mesh combines enough mesh density to capture the variations in the stress patterns, thus guaranteeing stable results^42^ when considering that a high-quality mesh should have a high level of homogeneity in the size of its elements^43^ in order to assure that the subsequent statistical analyses are not affected by the size of each element. The number of nodes and elements of each jaw model can be found in Table S1.

The thickness of the model was assumed to be constant throughout the mandible and was obtained from the individual average of three measurements; THK1) mandibular width at the first premolar, THK2) mandibular width at the mid-point of the mesio-distal length of the molar and premolar series and THK3) mandibular width at the posterior end of the molar series (Table S1 shows the thickness used in each model). Isotropic, homogeneous and linear elastic properties were assumed based on bovine harversian bone data: E [Young´s modulus] = 10 GPa and v [Poisson ratio] = 0.4^44^, even though it has been shown that this value is not essential in a comparative analysis^45^.

### Bite conditions

**Figure 1:**
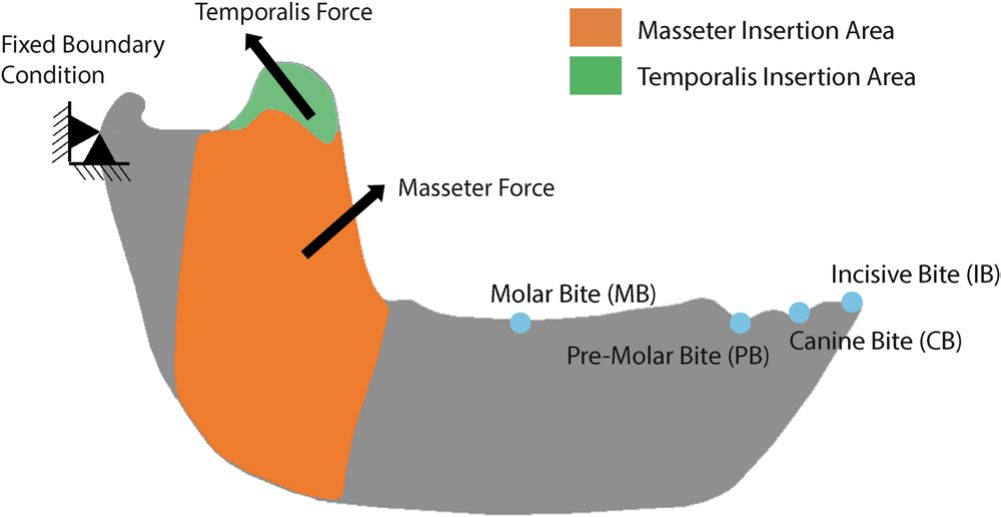
Free-Body Diagram of the biomechanical problem with the boundary conditions, the muscular forces, the area of insertion and the Bite position in IB: incisive bite; CB: canine bite; PB: premolar bite and MB: molar bite.

Boundary conditions were defined and placed to represent the loads and fixed displacements that the jaw experiences during feeding. The first boundary condition fixes the mandible at the most posterior point of the condyle at the level of the contact points with the mandibular fossa of the cranium (Fig. 1). The second boundary condition simulated biting and was positioned at four different tooth positions, describing four different cases:

1. IB (incisive bite): At the buccal alveolar margin of the incisive.
2. CB (canine-bite): At the centre of the canine at the level of the alveolar margin
3. PB (pre-molar bite): Between the most distal Premolar and the first Molar at the level of the alveolar margin.
4. MB (molar bite): At the centre of M1.

### Muscle Forces: Scaling the forces

Due to the fact that specimens analysed here exhibit significant size differences, the forces applied in the FEA models were scaled using the quasi-homothetic transformation proposed by Marcé-Nogué *et al*.^46^ in order to allow the comparison between models. Scaling the forces allows an appropriate comparison between stress results, although it is important to notice that this is not correcting for any allometric effects that might be involved. Size and function are intertangled because many biological variables correlate with size, thus correcting for allometry would remove some of the differences in shape related to function, which are aspects we are interested on. Therefore, when interpreting the FEA results, it is relevant to bear in mind that in spite of the scaling, allometry has to be considered. The focus of the present study was the comparison between these models, hence an arbitrary force of 1 N was applied in both muscles insertion areas and muscle force was assumed proportional to the insertion area in the reference model (i.e. *H. sapiens*). For the rest of the models under study, a proportional force based on their size differences was calculated also assuming that the muscle attachment is proportional to the muscle force (Table S2).

Since our goal was to carry out a comparative analysis similar to Serrano-Fochs *et al*.^29^, we were not interested in the *in vivo* force values or to validate our results against experimental data^47^. Instead we analyse stress levels under equivalent loads. For this reason, the proposed equation 1 allows comparisons between stress distributions when differences in size are affecting the model for plane stress changing the values of the forces applied. *H. sapiens* was used as a reference model with a value of F = 1 N.

1 $${F}_{B}=(\sqrt{\frac{{S}_{B}}{{S}_{A}}})(\frac{{t}_{B}}{{t}_{A}}){F}_{A}$$

where S_A_ was the area of a reference model, S_B_ the area of a scaled model, t_A_ was the thickness of a reference model and t_B_ the thickness of a scaled model. The value of the total force was distributed between the masseter and the temporalis in function of the insertion area (Table S2).

### Analysis of von Mises Stress

When comparing different models, von Mises stress distribution is the most adequate criterion for predicting the yield of a ductile materials when isotropic material properties are used in cortical bone^48^. The observed differences in stress distribution patterns may provide clues on different aspects of the diet of the analysed species. Assuming that more robust or stronger jaws would be needed for processing harder food items, lower values of stress should be expected. Conversely, weaker mandibles should be expected (i.e. showing higher stress levels) for species consuming softer diets.

A quantitative single measurement of the relative strength of the structure under study was preferred to summarise the strength of the whole model. The most common approach is the computation of the average von Mises stresses of the various FEM considered. Even though this approach has been used previously in palaeobiological studies^28, 49, 50^, we apply here the recently proposed weight-meshed values (mesh-weighted average mean (MWAM) and mesh-weighted median (MWM)) and the quasi-ideal meshes (QIM) and its percentile values (M(25%), M(50%), M(75%) and M(95%)) as a basis for our analysis^41^. The use of box-plots for the stress follows the idea of previous works^49^. Nonetheless in the present study, the use of a QIM mesh, facilitated the comparison between models and included the corrections to account for the non-uniformity of the mesh. In order to ensure a QIM, we computed the required errors^41^ to be sure that they fulfil the requirements (Percentages of error PEofAM < 2% and PEofM < 5%) (see supplementary note 1 for definitions).

### Phylogenetic signal and ancestral state reconstruction

**Figure 2:**
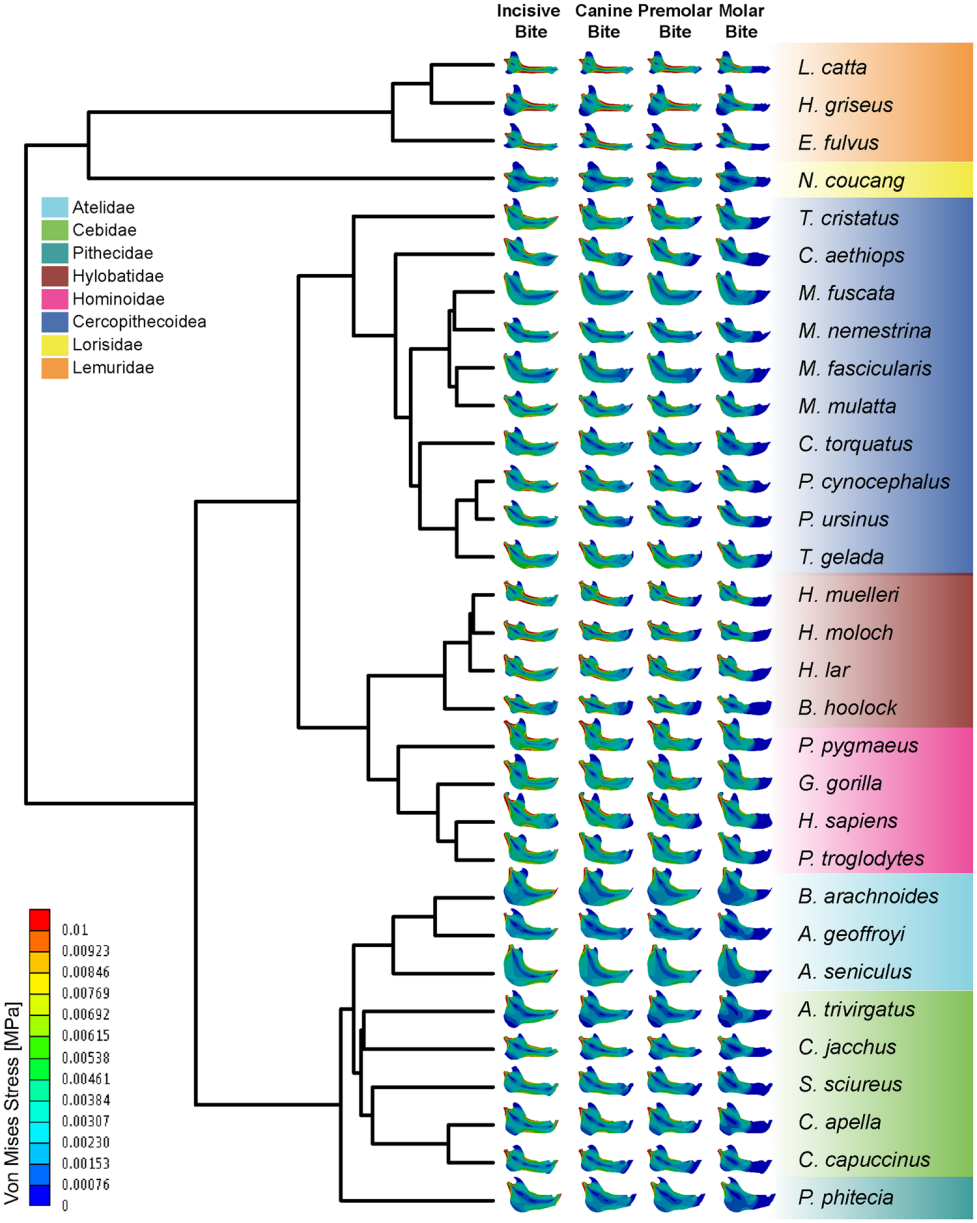
Phylogenetic tree and von Mises Stress distribution for each specimen under the four different bite cases: IB: incisive bite; CB: canine bite; PB: premolar bite and MB: molar bite. Phylogenetic tree from http://10ktrees.nunn-lab.org/.

Phylogenetic signal can be defined as the tendency for related species to resemble each other, more than they resemble species drawn at random from a phylogenetic tree^51^. A recursive analysis using 1000 phylogenies obtained from 10ktrees (http://10ktrees.nunn-lab.org/) was performed (Fig. 2). These phylogenies were sampled from a Bayesian phylogenetic analysis of molecular data for eleven mitochondrial and six autosomal genes available in GenBank^52^. In this study, phylogenetic signal was calculated using Pagel’s λ^53^ in the R package ‘geiger’^54^; for λ = 1 the tree is unchanged and the model is equivalent to Brownian motion, while for λ = 0 the tree becomes a star phylogeny and the model is equivalent to completely independent random walks, while values between 0 < λ < 1 provide an intermediate range where the correlations are weaker than expected.

In addition, the stress values of the models for the four biting cases were used to estimate the ancestral states for internal nodes using maximum likelihood and then by interpolating the states along the branches of the tree^51^ in the R package ‘phytools’^55^. This approach was applied to get insight about the possible evolution of mandibular stiffness in the order Primates.

### Statistical analysis

Statistical analyses were performed in Past v. 3.15^56^. By applying a Shapiro-Wilk test we checked the normality assumption for the stress values for each group (for both hardness and diet). The results showed that none of them followed a normal distribution and that median values must be used instead. However, we also present the average values because they also support the hypothesis herein discussed.

Two-Way PERMANOVA tests were performed to test for differences between diet categories, food hardness and stress values. This statistic was preferred because it is a non-parametric test of significant difference between two or more groups, based on any distance measure^57^. We tested the null hypothesis that there is no significant effect of the diet on the stiffness of the jaw, that there is no significant effect of the hardness of the consumed food on mandibular stiffness and that the interaction between diet and the hardness of the food do not have a significant effect on the stiffness of the mandible. The Two-Way PERMANOVA test was used to perform multiples comparisons between the non-normal data and applied to each bite case using as groups the diet and hardness categories defined in Table 1. Euclidean distances were used as similarity measure^57^ and 9999 permutations were performed.

As indicated above, we used as measure of stress the mesh-weighted values MWAM and MWM in the percentiles 25%, 50%, 75% and 95%. The highest value of the boxplot was not considered because an unusually high stress appears where the boundary conditions are set as a simple support. These stresses are artificially inflated by the constraints imposed on the model due to a numerical singularity^43^. This numerical singularity is a consequence of the mathematical approach involved, and it is not related to any biological process, therefore in those areas stresses have the tendency to increase towards infinity; thus, the results of these areas should not be considered.

## Results

### Von Mises Stress

Although Molar Bite and Premolar Bite exhibited an important area of lower stress in the region between the bite position and the frontal part of the body because no force was applied there, all mandibles showed a high level of stress at the mandibular notch, and from the condyle through the ramus in a descending direction (Fig. 2). From a general perspective, it seems that Lemuridae, Hylobatidae and Hominoidae showed areas with higher stress whereas Atelidae and Cebidae exhibited lower stresses.

**Figure 3:**
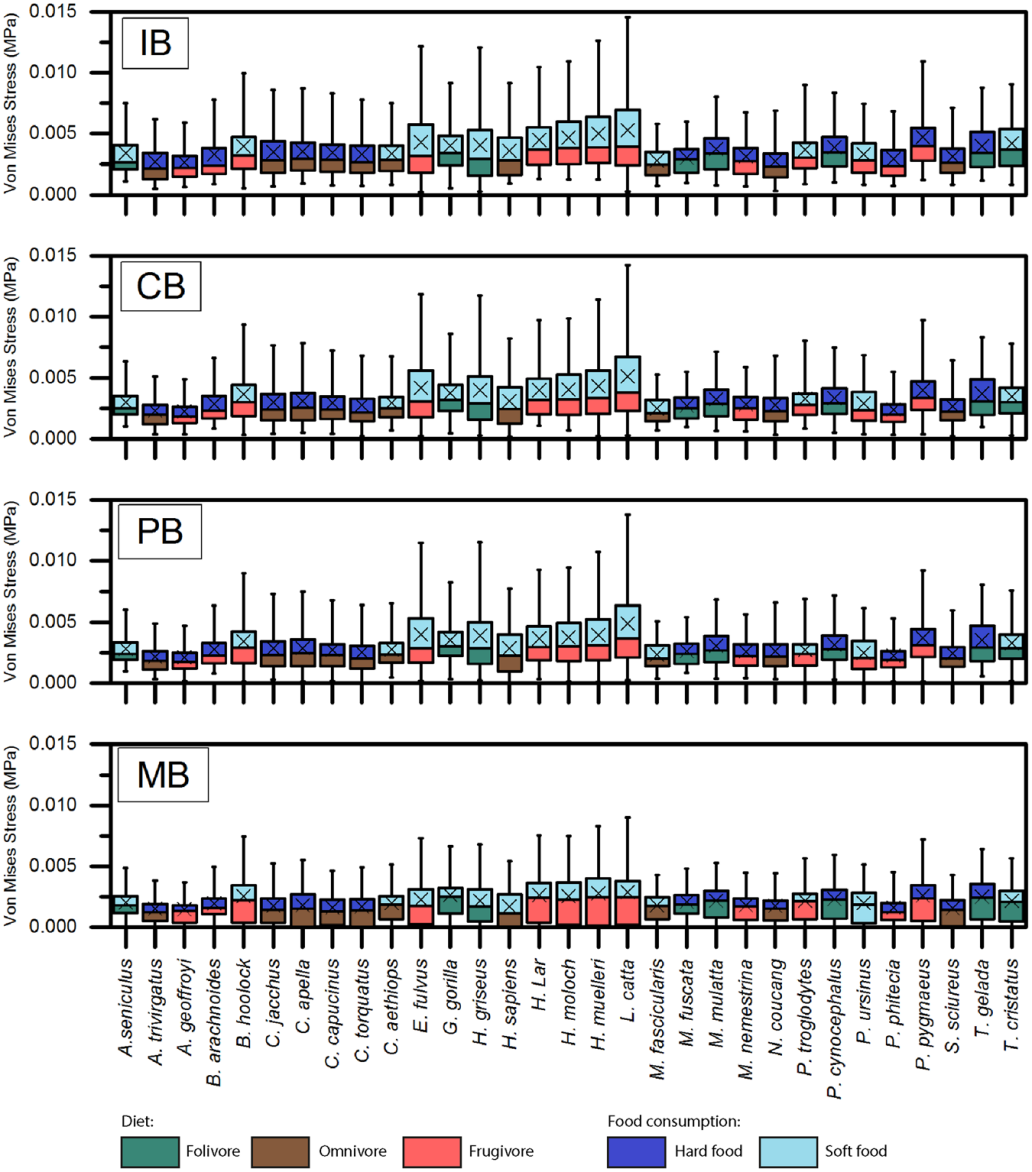
Box-plots of Von Mises stress distributions when QIM is assumed for the primates’ jaw analysed in the four biting cases. IB: incisive bite; CB: canine bite; PB: premolar bite and MB: molar bite.

In order to better understand the intensity of these stresses quantitative methods were applied: Fig. 3 shows the stress distribution of the QIM in boxplots. For instance, the visual representation of the stress distribution for each mandible is a useful indicator for comparative inference on their biomechanical behaviour because these stress patterns can be interpreted as a sign of relative strength, with specimens with higher stresses being consequently weaker. The values of MWAM, MWAM, the quartiles of the boxplots of stress and the PEofAM and PEofM (percentages of error used to define the QIM) can be found in the Supplementary information (Tables S3, S4, S5 and S6). They were calculated for the four biting cases.

### Phylogenetic and Statistical analysis

Results shown in Table 2 indicate that there was no noticeable phylogenetic signal as measured using Pagel’s λ for all the stress variables considered here.

**Table 2:** Pagel’s λ of the four biting cases calculated for the variables: MWAM, MWM, M(25%), M(50%), M(75%) and M(95%).

	MWAM	MWM	M(25%)	M(50%)	M(75%)	M(95%)
IB	0.67	0.66	0.00	0.73	0.54	0.00
CB	0.63	0.68	0.50	0.71	0.58	0.41
PB	0.27	0.61	0.59	0.00	0.66	0.60
MB	0.56	0.52	0.65	0.70	0.40	0.65

The Two-Way PERMANOVA (N = 9999) test rejected the null hypothesis proposed to test the effect of diet and hardness of the food on mandibular stiffness in each one of the bite cases and for the different stress parameters analysed. All the von Mises stress parameters presented p-values < 0.05. On the other hand, the interaction of diet and food hardness showed no significant effects with all the p-values > 0.05 (Table 3). For further statistical details see Tables S7, S8 and S9.

**Table 3:** p-values for the Two-Way PERMANOVA for the four biting cases (IB: Incisive Bite, CB: Canine Bite, PB: Premolar Bite and MB: Molar Bite) when analysing MWAM, MWAM and all the percentiles together.

	MWAM	MWM	M(25%), M(50%), M(75%) and M(95%)
IB			
Diet	0.0041	0.0082	0.0067
Hardness	0.0019	0.0076	0.0011
Interaction	0.3993	0.2112	0.2046
CB			
Diet	0.0024	0.0005	0.0116
Hardness	0.0009	0.0007	0.0008
Interaction	0.2789	0.1322	0.1652
PB			
Diet	0.0151	0.0084	0.0172
Hardness	0.0013	0.0022	0.0016
Interaction	0.6727	0.6280	0.2797
MB			
Diet	0.0079	0.0125	0.0006
Hardness	0.0023	0.0241	0.0005
Interaction	0.8343	0.5243	0.1570

**Figure 4:**
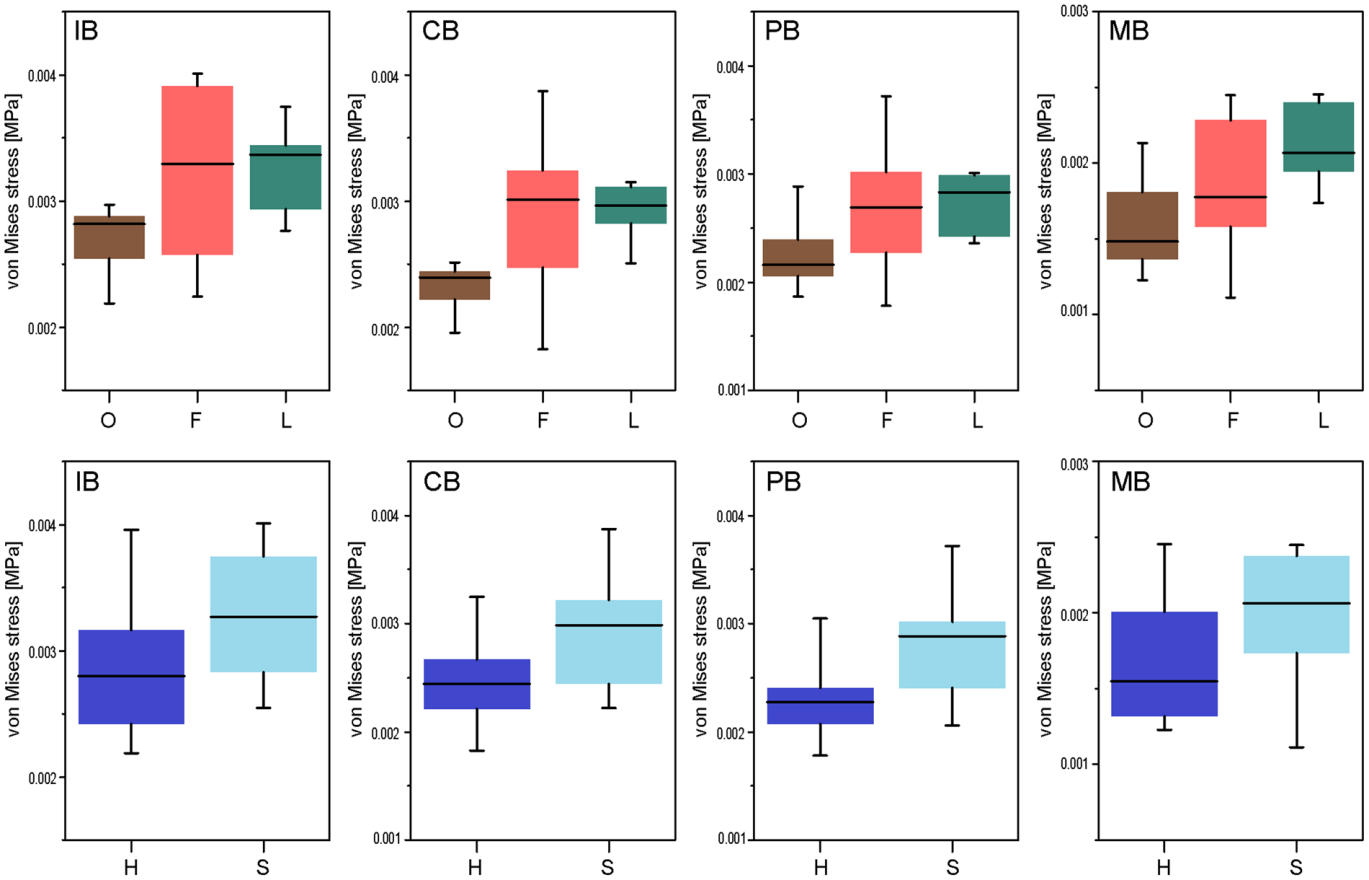
Box-plots of the MWM values of all species grouped by hardness of ingesta (H: hard eaters; S: soft eaters) and by dietary categories (O: omnivore; F: frugivore and L: folivore). IB: incisive bite; CB: canine bite; PB: premolar bite and MB: molar bite. The median is the middle line of the box and whiskers represent the range.

Regarding the food hardness categories, our results clearly indicate that hard-food eaters have stiffer jaws as compared to soft-food consumers. When comparing dietary categories, we found some patterns in MWM values as visualized in Fig. 4: Folivores exhibited the weakest jaws, whereas omnivores show lower stress values, thus implying that they have stiffer mandibles. Frugivores present a wider range of values.

Figure S1 in the supplementary information correspond to boxplots of the percentiles 75% and 95% and the MWAM. It is interesting that for the maximum von Mises stress values observed in the jaws –which are those responsible of a hypothesized failure of the mandible- the trend is the same for the diet categories and food hardness.

**Figure 5:**
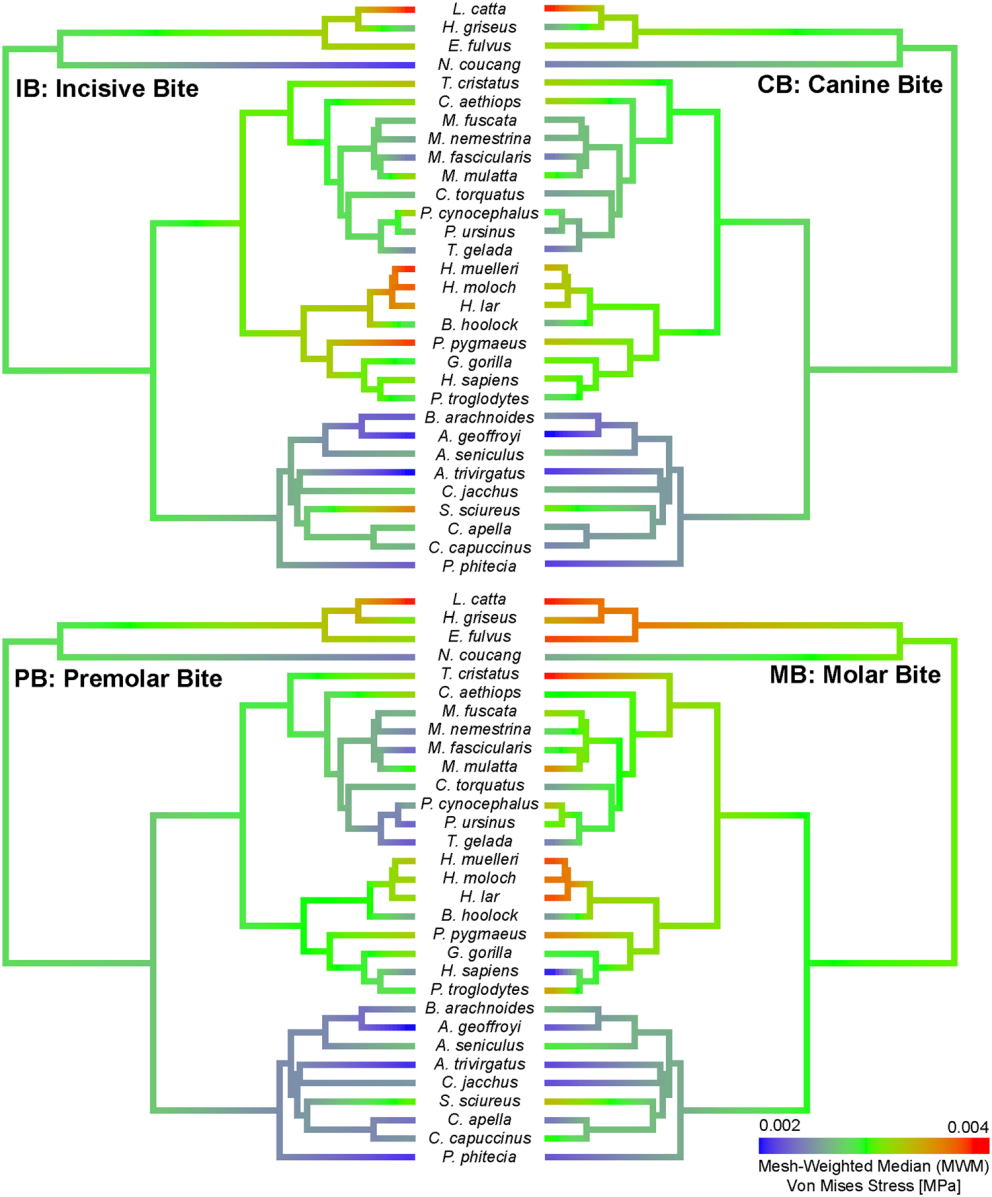
MWM values for each species mapped on the phylogeny for the four biting scenarios. The values at nodes and branches were reconstructed using a maximum-likelihood ancestral character estimation method based on a Brownian motion model of evolution. The colour ranges from red representing higher average stress values, to blue, representing lower stress values.

Figure 5 depicts the results of the ancestral state reconstruction for the stress median measurements (MWM). These values were mapped on the phylogeny using a maximum-likelihood ancestral character estimation method based on a Brownian motion model of evolution. The results show that the ancestral condition seems to exhibit an intermediate position between the two possible biomechanical extremes. Certain lineages seem to have evolved towards more resistant mandibles (e.g. platyrrhines [excepting *Saimiri*] and the Papionini tribe), while others seem to have evolved towards less stiffer jaws (e.g. the Hylobatidae and *Lemur catta*). Figures S2, S3 and S4 in the supplementary information depicts the ancestral state reconstruction for the values of stress percentiles 75% and 95% and the MWAM.

## Discussion

Studying the degree to which mandibular form is associated with its biomechanical function provides a unique chance to analyse dietary adaptations, as well as potentially providing a tool to reconstruct the feeding behaviour of extinct species. It has been proposed that a species’ dietary niche occupation should be reflected in its functional morphology^58^.

Based on the results obtained for the higher stress values (M(75%) and M(95%)) as well as for the average values (MWAM and MWM), we found that there is robust evidence to accept that hard-eaters have stiffer jaws when compared to primates that consume softer foods. In addition, we also found that there is sufficient support to suggest that folivores have the weakest mandibles among primates whereas omnivores have the strongest ones. The lower values represented by M(25%) showed that there is some p-values that are not significant (e.g. Molar Biting) although they are not characteristic of the strength of the jaw and, as a consequence, not used in our interpretation. These results are consistent with previous research on armadillos^29, 41^, marine mammals^59^, Artiodactyla and Perissodactyla^27^ and Carnivora^60^. The latter studies used FEA to test mandibular stiffness in relation to diet and feeding classification and yielded significant relationships.

From a biomechanical perspective, stiffer jaws exhibit larger areas of lower stress values along with lower peak values as compared to “weaker” jaws at same biting force (scaled according to the size of the jaw). For higher bite forces, the stress values in the mandible increase proportionally to reach the threshold where bone can break. If loaded increasingly, “weaker” jaws would reach the failure load earlier than the stiffer ones. Consequently, stiffer jaws allow the consumption of food items that might require higher bite forces, or alternatively that need more local and focalised force implementation. The present results also showed – from a qualitative point of view- that primates exhibiting higher consumption of harder foods (e.g. seeds and/or animals) require a stiffer jaw to withstand the higher forces generated when processing these harder items.

The higher the forces required to fracture foods or their protective structures, as well as the more frequently these forces have to be transmitted, the stronger the lower jaw has to be built in order to preserve its structural integrity. The types of food items that impose high mechanical demands are those that are particularly hard and/or tough and those that require extensive repetitive chewing. The mastication of foods that are especially hard such as seeds habitually implies a greater recruitment of both working-side and especially balancing-side masticatory muscles^61–63^. These results are also in agreement with previous experimental studies of mandibular development in animals fed with different diets. Research carried out on different mammals including primates has shown that durophagy (i.e., a hard, strong, or tough ingesta) elicits greater cortical bone modelling and remodelling in the mandibular corpora of growing animals when compared to control subjects fed on a softer diet^12, 64, 65^. Consequently, species that usually bite and/or chew on hard items exhibit mandibular corpora, which are more resistant to torsion and parasagittal bending^66^.

As expected, the observed stress distribution in the FE models are related with the geometry/morphology of the jaw. Different shapes result in different stress patterns. For example, more pointed condylar geometries along with more acute mandibular angles favoured higher peak stresses as compared to smoother and flatter geometries. On the other hand, wider mandibular corpora showed more extended areas of lower stress. For instance, *M. nemestrina* is typically classified a hard food eater and it is the one with the most obtuse angle between the corpus and the ramus of the jaw in this study, thus showing lower stresses than the other macaques. The geometry of the mandibular body for both Atelidae and Cebidae, which are all considered hard food eaters except for *A. seniculus*, is wider than the geometry indicative of families dominated by soft food consumers such as Lemuridae and Hylobatidae. Previous evidence for the proposed link between mandibular form and diet have shown mixed or inconsistent results, thus suggesting that there is no straightforward relationship between mandibular shape and diet (e.g. refs 67 and 68). This archetypal model assumes that the morphology of the feeding apparatus of an animal corresponds to what it eats, thus being expected that the adaptation to different foods would account for the majority of the observed interspecific morphologic diversity. However, studies have found different levels of predictive power depending on the functional and morphological data employed^67, 69^. Furthermore, it seems that the capacity to relate the shape of a given mandibular corpus with a particular diet depends on the clade under study^67–69^. Several studies have focused on the primate mandible and its functional adaptation to different ingesta (see Ross *et al*.^70^ for a review). More recently by applying geometric morphometric techniques, it has been found that in primates there is a weak but significant impact of diet on mandibular shape diversity when this order is analysed as a whole but not in anthropoids and catarrhines when tested separately, because it seems that these clades showed allometric changes in shape that are unrelated to diet^69^. However, there are few studies that have tried to elucidate function based on an explicit biomechanical framework. We thus consider our results highly relevant because they suggest an association between mandibular biomechanical performance, food hardness and general dietary categories that can be inferred using the techniques suggested here rather than from sole morphology and morphometrics.

The ancestral state reconstruction for the median stress values (MWM) was consistent with the pattern described above. Certain lineages seem to have evolved towards more resistant mandibles such as the Papionini tribe and most platyrrhines, while others seem to have maintained or even evolved towards less stiffer mandibles such as *Lemur catta* and the Hylobatidae for all the analysed biting scenarios. *Lemur catta* showed the least resistant mandible among all species analysed. The other members of Lemuridae considered here only show similarities to *L catta* during molar biting, as indicated by high stress values. Interestingly, in the ape clade the ancestral condition seem to have been an intermediate condition that subsequently evolved towards less stress resistant mandibles in gibbons and *Pongo* while the remaining extant apes retained an intermediate biomechanical condition. The results found in *Pongo* are surprising because orangutans eat fruits containing hard seeds and therefore one would expect a more load resistant morphology. It is intriguing that during incisive and molar biting the orangutan showed significantly lower load resistance values as compared to the other hominids. This species should certainly be subject to more specialized biomechanical analysis in order to better understand this result. Our results are also in agreement with the cumulative evidence that has shown that *Homo sapiens* is an efficient producers of bite force particularly during molar biting^71^ and may be considered a rather chewing adapted species.

## Conclusions

The present study has provided robust evidence to support the hypothesis, that among primates, hard food eaters have more load resistant (i.e. stiffer) mandibles when compared to those that rely on softer ingesta. In addition, we found that folivorous primates have the least load resident (i.e. weakest) mandibles within the primate order whilst omnivores exhibit the strongest jaws. Interestingly, species that usually bite and/or chew on hard items exhibit mandibular corpora, which are more resistant during the analysed loading cases. The present results are expected to contribute to a better understanding of the relationship between mandibular morphology and mechanical performance because they show how the relationship between mandibular biomechanical performance, mandibular form, food hardness and dietary categories can be elucidated using biomechanical techniques rather than focusing exclusively on morphology.

## Electronic supplementary material

Supplementary information
