# Supplementary material for: A biomechanical approach to understand the ecomorphological relationship between primate mandibles and diet
Source: Sci Rep. 2017 Aug 21;7:8364. doi: 10.1038/s41598-017-08161-0 (PMC5567063; doi:10.1038/s41598-017-08161-0)

A biomechanical approach to understand the ecomorphological relationship between primate mandibles and diet.

Jordi Marcé-Nogué[1], Thomas A. Püschel[2] and Thomas M. Kaiser[1]

[1]Centrum für Naturkunde, University of Hamburg, Martin-Luter-King-Platz 3 20146 Hamburg (Germany)

[2]School of Earth and Environmental Sciences, University of Manchester, Oxford Road, M13 9PL, UK.

*corresponding author: jordi.marce.nogue@uni-hamburg.de

Supplementary Note 1: MWAM, MWM, PEofAM and PEofM definitions

MWAM: Mesh-Weighted Arithmetic Mean of Stress Distribution:

The arithmetic mean is calculated by summing all the individual observations or items of a sample and dividing this sum by the number of items in the sample. In FEA results of stress, the Arithmetic Mean (AM) would be the sum of the value of the Von Mises stress (σ_{VM}) of each element divided by the number of elements of the mesh (equation 1).

$$AM = \frac{\sum_{i=0}^{n} \sigma_{VM}}{n}$$

(Equation 1)

The Mesh-Weighted Arithmetic Mean (MWAM) corresponds to the sum of the value of the Von Mises stress for each element multiplied by its own area (A) and divided by the total area (equation 2). This value is equivalent to the division of the arithmetic mean of the product of stress and area by the arithmetic mean of the area, which is easier to calculate and does not require the correction of the weight element by element.

$$MWAM = \frac{\sum_{i=0}^{n}\left(\sigma_{VM}^{i} \cdot A^{i}\right)}{\sum_{i=0}^{n} A^{i}} = \frac{\frac{\sum_{i=0}^{n}\left(\sigma_{VM}^{i} \cdot A^{i}\right)}{n}}{\frac{\sum_{i=0}^{n} A^{i}}{n}} = \frac{AM\left(\sigma_{VM}^{i} \cdot A^{i}\right)}{AM\left(A^{i}\right)}$$

(Equation 2)

MWM: Mesh-Weighted Median of Stress Distribution:

The median is the middle measurement of any set of sorted data. In the case of FEA, the median would be the value separating the higher half from the lower half of the values of Von Mises Stress recorded in each element of the mesh after they have been ordered.

Here the Mesh-Weighted Median (MWM) of Stress Distribution has been defined as the division of the median of the product of stress and area by the median of the area (Equation 3), based in the formulation presented in Equation 2.

$$MWM = \frac{median\left(\sigma_{VM}^{i} \cdot A^{i}\right)}{median\left(A^{i}\right)}$$

(Equation 3)

PEofAM and PEofM: Percenntage Error:

Two indicators were proposed to evaluate whether a mesh is uniform enough to use the raw stress data for statistical analysis: the Percentage Error of the Arithmetic Mean (PEofAM) and the Percentage Error of the Median (PEofM). These two indicators evaluate the difference between the non-weighted value and the weighted value of mean and median (PEofAM in equations 4 and PEofM in equation 5),

$$PEofAM = \left(\frac{MWAM - AM}{MWAM}\right) \times 100$$

(Equation 4)

$$PEofM = \left(\frac{MWM - M}{MWM}\right) \times 100$$

(Equation 5)

If the mesh of the model is close to an ideal fine uniform mesh the non-weighted and the weighted indicators should be equal. If the error is lower than a certain threshold the mesh can be considered a QUIM, and quantitative and statistical analysis of the FEA data can be computed without any corrections and the percentile values (M(25%), M(50%), M(75%) and M(95%)) can be computed.

Table S1 FEA characteristics of the models: Number of nodes, elements and jaw thickness. The thickness of the model was assumed to be constant throughout the mandible and this value was obtained from the individual average of this three measurements: THK1, THK2 and THK3

Species	FEA Model		Jaw thickness			
	Nodes	Elements	THK1 [mm]	THK2 [mm]	THK3 [mm]	Average [mm]
A. geoffroyi	91999	91245	5.49	5.28	5.09	5.29
A. trivirgatus	80375	79697	3.48	3.12	3.24	3.28
A.seniculus	93217	92495	8.37	7.76	7.05	7.73
B. arachnoides	103695	102949	7.31	7.40	9.33	8.01
B. hoolock	54421	53828	6.76	4.85	4.88	5.50
C. aethiops	75989	75310	5.97	5.85	7.04	6.29
C. apella	94325	93558	7.60	6.63	8.51	7.58
C. capucinus	77262	76550	6.28	5.44	6.36	6.03
C. jacchus	75875	75183	2.61	2.10	2.17	2.29
C. torquatus	203334	202203	6.31	5.07	5.46	5.61
E. fulvus	58477	57745	4.36	4.00	3.64	4.00
G. gorilla	75524	74842	22.13	18.16	24.78	21.69
H. griseus	79488	78706	4.62	4.34	4.54	4.50
H. Lar	90901	90114	8.16	6.08	7.92	7.39
H. moloch	89901	89099	7.79	6.80	7.15	7.25
H. muelleri	87710	86862	7.17	5.61	5.66	6.15
H. sapiens	70714	70033	16.23	15.52	14.32	15.36
L. catta	82693	81852	3.63	3.52	3.64	3.60
M. fascicularis	78436	77746	15.34	9.93	11.15	12.14
M. fuscata	71866	71222	12.30	10.33	10.28	10.97
M. mulatta	77046	76325	10.20	8.08	9.36	9.21
M. nemestrina	83626	82885	11.68	9.95	8.72	10.12
N. coucang	77445	76775	4.72	4.07	4.06	4.28
P. cynocephalus	97881	97087	14.07	12.21	13.07	13.12
P. Pithecia	81868	81192	5.13	4.64	4.60	4.79
P. pygmaeus	78952	78243	17.47	13.03	15.14	15.21
P. troglodytes	65860	65219	16.25	13.51	16.23	15.33
P. ursinus	81796	81036	13.84	11.55	11.64	12.34
S. sciureus	73748	73067	3.55	2.80	3.60	3.32
T. cristatus	97232	96412	7.10	6.05	6.20	6.45
T. gelada	99368	98530	11.36	10.74	12.31	11.47

Table S2. Parameters needed for Equation 1 to define the scaling of the forces (Average Thickness, Masseter and Temporalis surface, Model Area) and the values of the scaled force.

Species	Average Thickness [mm]	Masseter Surface [mm^2]	Temporalis Surface [mm^2]	Model Area [mm^2]	Total Force [N]	Masseter force [N]	Temporalis force [N]
A. geoffroyi	5.29	593.70	50.93	728.94	0.1401	0.1291	0.0111
A. seniculus	7.73	1106.50	33.69	2159.30	0.3525	0.3421	0.0104
A. trivirgatus	3.28	214.70	27.87	455.90	0.0688	0.0609	0.0079
B. arachnoides	8.01	1222.90	106.93	2357.10	0.3819	0.3512	0.0307
B. hoolock	5.50	133.24	15.96	536.75	0.1250	0.1116	0.0134
C. aethiops	6.29	275.03	20.64	762.95	0.1705	0.1586	0.0119
C. apella	7.58	388.70	17.48	957.04	0.2302	0.2203	0.0099
C. capucinus	6.03	292.94	17.55	780.59	0.1653	0.1560	0.0093
C. jacchus	2.29	96.84	6.73	193.52	0.0313	0.0293	0.0020
C. torquatus	5.61	529.46	25.66	1181.90	0.1895	0.1807	0.0088
E. fulvus	4.00	265.75	17.11	592.57	0.0956	0.0898	0.0058
G. gorilla	21.69	5698.20	711.58	12241.00	2.3559	2.0944	0.2615
H. griseus	4.50	206.22	30.30	456.32	0.0944	0.0823	0.0121
H. Lar	7.39	264.09	38.97	920.07	0.2200	0.1917	0.0283
H. moloch	7.25	266.05	49.69	908.44	0.2144	0.1807	0.0337
H. muelleri	6.15	321.73	25.85	881.87	0.1792	0.1659	0.0133
H. sapiens	15.36	1815.10	192.24	4470.30	1.0080	0.9115	0.0965
L. catta	3.60	178.86	11.71	469.50	0.0765	0.0718	0.0047
M. fascicularis	12.14	1156.00	85.32	1931.00	0.5237	0.4877	0.0360
M. fuscata	10.97	888.20	90.47	2204.10	0.5056	0.4589	0.0467
M. mulatta	9.21	723.45	81.71	1744.30	0.3778	0.3394	0.0383
M. nemestrina	10.12	1059.70	108.54	2685.50	0.5147	0.4669	0.0478
N. coucang	4.28	216.10	15.58	451.17	0.0893	0.0833	0.0060
P. cynocephalus	13.12	1645.90	162.61	3951.00	0.8094	0.7366	0.0728
P. Pithecia	4.79	392.13	20.46	791.54	0.1323	0.1257	0.0066
P. pygmaeus	15.21	1968.60	208.64	4953.90	1.0510	0.9503	0.1007
P. troglodytes	15.33	1601.10	137.57	4115.40	0.9655	0.8891	0.0764
P. ursinus	12.34	1925.70	258.50	5127.80	0.8677	0.7650	0.1027
S. sciureus	3.32	109.40	8.21	267.73	0.0533	0.0496	0.0037
T. cristatus	6.45	395.59	34.86	986.33	0.1989	0.1828	0.0161
T. gelada	11.47	1806.60	63.28	4033.60	0.7152	0.6910	0.0242

Table S3 Values of von Mises Stress for IB (Incisive Bite) and error percentages of the respective QIMs.

Species	MWAM [Mpa]	MWM [Mpa]	M(25%) [Mpa]	M(50%) [Mpa]	M(75%) [Mpa]	M(95%) [Mpa]	PeofAM	PEofM
A. geoffroyi	0.002651	0.002243	0.001475	0.002184	0.003201	0.005908	1.08	2.61
A. seniculus	0.003407	0.002763	0.002115	0.002670	0.004062	0.007502	0.81	3.36
A. trivirgatus	0.002673	0.002187	0.001316	0.002152	0.003411	0.006176	0.79	1.60
B. arachnoides	0.003289	0.002416	0.001731	0.002390	0.003859	0.007795	0.41	1.08
B. hoolock	0.004000	0.003268	0.002161	0.003233	0.004729	0.009962	0.37	1.06
C. aethiops	0.003349	0.002817	0.001960	0.002840	0.004027	0.007486	0.20	0.82
C. apella	0.003581	0.002912	0.002001	0.002952	0.004258	0.008731	0.17	1.34
C. capucinus	0.003473	0.002875	0.001896	0.002841	0.004141	0.008292	0.35	1.18
C. jacchus	0.003525	0.002972	0.001778	0.002817	0.004452	0.008644	0.47	1.40
C. torquatus	0.003342	0.002774	0.001796	0.002670	0.004008	0.007796	0.10	3.77
E. fulvus	0.004288	0.003322	0.001799	0.003183	0.005788	0.012148	0.45	4.17
G. gorilla	0.003997	0.003417	0.002423	0.003431	0.004827	0.009139	0.29	0.41
H. griseus	0.004063	0.003051	0.001604	0.002957	0.005303	0.012076	0.42	3.07
H. Lar	0.004468	0.003651	0.002462	0.003680	0.005515	0.010466	0.22	0.78
H. moloch	0.004691	0.003868	0.002517	0.003851	0.005995	0.010937	0.15	0.46
H. muelleri	0.005034	0.003998	0.002610	0.003892	0.006384	0.012650	0.25	2.68
H. sapiens	0.003654	0.002834	0.001648	0.002795	0.004669	0.009129	1.02	1.38
L. catta	0.005274	0.004011	0.002408	0.003932	0.006919	0.014554	0.11	1.97
M. fascicularis	0.002793	0.002544	0.001651	0.002494	0.003503	0.005814	0.47	1.94
M. fuscata	0.003015	0.002827	0.001801	0.002893	0.003736	0.006010	0.75	2.32
M. mulatta	0.003709	0.003348	0.002089	0.003348	0.004629	0.008039	0.40	0.01
M. nemestrina	0.003130	0.002720	0.001734	0.002752	0.003856	0.006768	0.13	1.18
N. coucang	0.002752	0.002288	0.001423	0.002286	0.003382	0.006899	0.75	0.08
P. cynocephalus	0.003880	0.003392	0.002338	0.003450	0.004729	0.008364	0.04	1.71
P. Pithecia	0.002946	0.002429	0.001576	0.002348	0.003639	0.006851	0.22	3.31
P. pygmaeus	0.004743	0.003959	0.002798	0.003979	0.005500	0.010922	0.56	0.52
P. troglodytes	0.003698	0.003011	0.002207	0.003058	0.004241	0.009015	0.11	1.58
P. ursinus	0.003298	0.002863	0.001830	0.002819	0.004216	0.007464	0.39	1.52
S. sciureus	0.003126	0.002616	0.001821	0.002596	0.003790	0.007142	0.09	0.75
T. cristatus	0.004204	0.003744	0.002394	0.003692	0.005411	0.009079	0.76	1.40
T. gelada	0.003977	0.003458	0.002299	0.003411	0.005149	0.008797	0.58	1.36

Table S4 Values of von Mises Stress for CB (Canine Bite) and error percentages of the respective QIMs.

Species	MWAM [Mpa]	MWM [Mpa]	M(25%) [Mpa]	M(50%) [Mpa]	M(75%) [Mpa]	M(95%) [Mpa]	Peof AM	PEo fM
A. geoffroyi	0.002239	0.001826	0.001330	0.001833	0.002622	0.004894	0.46	0.40
A. seniculus	0.002979	0.002556	0.002039	0.002503	0.003535	0.006338	0.31	2.08
A. trivirgatus	0.002309	0.001960	0.001199	0.001949	0.002778	0.005114	0.10	0.55
B. arachnoides	0.002888	0.002324	0.001679	0.002313	0.003512	0.006636	0.14	0.47
B. hoolock	0.003680	0.003007	0.001894	0.003012	0.004424	0.009373	0.49	0.16
C. aethiops	0.002949	0.002394	0.001789	0.002485	0.003448	0.006747	0.28	3.79
C. apella	0.003099	0.002515	0.001545	0.002533	0.003765	0.007843	0.61	0.71
C. capucinus	0.002964	0.002425	0.001644	0.002399	0.003457	0.007254	0.81	1.06
C. jacchus	0.003025	0.002471	0.001542	0.002381	0.003659	0.007641	0.48	3.65
C. torquatus	0.002788	0.002273	0.001441	0.002172	0.003308	0.006812	0.61	4.45
E. fulvus	0.004157	0.003150	0.001772	0.003035	0.005585	0.011858	0.34	3.67
G. gorilla	0.003732	0.003111	0.002315	0.003178	0.004430	0.008602	0.16	2.13
H. griseus	0.003971	0.002964	0.001595	0.002907	0.005142	0.011754	0.29	1.93
H. Lar	0.003981	0.003219	0.002008	0.003214	0.004953	0.009717	0.23	0.14
H. moloch	0.004055	0.003241	0.001991	0.003230	0.005253	0.009888	0.74	0.35
H. muelleri	0.004333	0.003364	0.002075	0.003349	0.005584	0.011403	0.57	0.45
H. sapiens	0.003143	0.002441	0.001257	0.002426	0.004226	0.008206	1.57	0.63
L. catta	0.005131	0.003874	0.002297	0.003811	0.006706	0.014265	0.06	1.62
M. fascicularis	0.002494	0.002220	0.001457	0.002129	0.003215	0.005250	0.20	4.07
M. fuscata	0.002733	0.002507	0.001668	0.002496	0.003358	0.005473	0.37	0.45
M. mulatta	0.003241	0.002934	0.001884	0.002857	0.004043	0.007125	0.15	2.64
M. nemestrina	0.002792	0.002475	0.001582	0.002481	0.003444	0.005880	0.13	0.21
N. coucang	0.002723	0.002259	0.001430	0.002247	0.003333	0.006807	0.81	0.54
P. cynocephalus	0.003365	0.002822	0.002050	0.002922	0.004117	0.007460	0.38	3.52
P. Pithecia	0.002397	0.001972	0.001409	0.001959	0.002815	0.005510	0.11	0.63
P. pygmaeus	0.004049	0.003247	0.002396	0.003314	0.004685	0.009732	0.94	2.07
P. troglodytes	0.003280	0.002683	0.001957	0.002747	0.003719	0.008038	0.14	2.39
P. ursinus	0.002893	0.002402	0.001479	0.002362	0.003865	0.006882	0.12	1.69
S. sciureus	0.002681	0.002166	0.001554	0.002200	0.003232	0.006426	0.41	1.56
T. cristatus	0.003461	0.002990	0.002132	0.003026	0.004165	0.007820	0.10	1.21
T. gelada	0.003680	0.003149	0.001948	0.003063	0.004870	0.008314	0.21	2.72

Table S5 Values of von Mises Stress for PB (Premolar Bite) and error percentages of the respective QIMs

Species	MWAM [Mpa]	MWM [Mpa]	M(25%) [Mpa]	M(50%) [Mpa]	M(75%) [Mpa]	M(95%) [Mpa]	Peof AM	PEo fM
A. geoffroyi	0.002131	0.001782	0.001213	0.001758	0.002496	0.004731	0.30	1.35
A. seniculus	0.002833	0.002460	0.001948	0.002419	0.003328	0.006028	0.36	1.68
A. trivirgatus	0.002191	0.001863	0.001118	0.001860	0.002636	0.004893	0.11	0.15
B. arachnoides	0.002775	0.002273	0.001628	0.002261	0.003278	0.006361	0.22	0.52
B. hoolock	0.002900	0.002274	0.001641	0.002276	0.004229	0.009002	0.59	1.34
C. aethiops	0.002894	0.002408	0.001715	0.002429	0.003280	0.006520	0.25	3.76
C. apella	0.002736	0.002278	0.001409	0.002322	0.003580	0.007467	0.81	0.89
C. capucinus	0.002553	0.002120	0.001425	0.002041	0.003193	0.006789	0.89	1.92
C. jacchus	0.002865	0.002388	0.001412	0.002329	0.003452	0.007312	0.42	2.47
C. torquatus	0.002803	0.002286	0.001229	0.002372	0.003045	0.006385	0.71	3.74
E. fulvus	0.003980	0.003016	0.001693	0.002896	0.005317	0.011475	0.33	3.99
G. gorilla	0.003563	0.002985	0.002250	0.003016	0.004179	0.008256	0.02	2.09
H. griseus	0.003895	0.002928	0.001603	0.002854	0.005005	0.011538	0.31	2.54
H. Lar	0.003682	0.002974	0.001903	0.002962	0.004641	0.009285	0.22	0.39
H. moloch	0.003778	0.003020	0.001833	0.003015	0.004962	0.009435	0.88	0.16
H. muelleri	0.003992	0.003113	0.001898	0.003128	0.005212	0.010721	0.65	0.48
H. sapiens	0.003467	0.002883	0.001005	0.002922	0.004005	0.007731	1.54	0.11
L. catta	0.004896	0.003717	0.002135	0.003671	0.006377	0.013783	0.02	1.22
M. fascicularis	0.002369	0.002071	0.001395	0.002021	0.003091	0.005079	0.06	2.39
M. fuscata	0.002643	0.002422	0.001600	0.002396	0.003250	0.005398	0.28	1.07
M. mulatta	0.003064	0.002790	0.001723	0.002700	0.003866	0.006836	0.02	3.20
M. nemestrina	0.002595	0.002211	0.001446	0.002204	0.003210	0.005646	0.12	0.33
N. coucang	0.002638	0.002157	0.001417	0.002159	0.003216	0.006571	0.73	0.05
P. cynocephalus	0.002733	0.002363	0.001921	0.002393	0.003909	0.007139	0.36	2.97
P. Pithecia	0.002251	0.001884	0.001301	0.001870	0.002641	0.005337	0.02	0.79
P. pygmaeus	0.003768	0.003047	0.002182	0.003108	0.004404	0.009223	0.95	2.02
P. troglodytes	0.003184	0.002689	0.001444	0.002768	0.003222	0.006874	0.47	1.27
P. ursinus	0.002495	0.002055	0.001176	0.002060	0.003465	0.006183	0.23	0.24
S. sciureus	0.002433	0.002029	0.001375	0.002038	0.002968	0.005964	0.15	0.42
T. cristatus	0.003307	0.002829	0.002036	0.002862	0.004014	0.007563	0.07	1.16
T. gelada	0.003512	0.003010	0.001838	0.002918	0.004703	0.008059	0.04	3.06

Table S6 Values of Von Mises Stress for MB (Molar Bite) and error percentages of the respective QIMs

Species	MWAM [Mpa]	MWM [Mpa]	M(25%) [Mpa]	M(50%) [Mpa]	M(75%) [Mpa]	M(95%) [Mpa]	Peof AM	PEo fM
A. geoffroyi	0.001460	0.001265	0.000393	0.001313	0.001822	0.003692	0.44	3.73
A. seniculus	0.002047	0.001735	0.001191	0.001789	0.002563	0.004853	0.19	3.10
A. trivirgatus	0.001521	0.001229	0.000556	0.001228	0.001907	0.003826	0.54	0.04
B. arachnoides	0.001982	0.001581	0.001103	0.001583	0.002369	0.004965	0.90	0.11
B. hoolock	0.001720	0.001110	0.000413	0.001145	0.003463	0.007443	1.65	4.49
C. aethiops	0.001849	0.001483	0.000687	0.001530	0.002548	0.005160	0.07	2.96
C. apella	0.001652	0.001245	0.000085	0.001284	0.002681	0.005519	1.24	3.12
C. capucinus	0.001711	0.001364	0.000200	0.001363	0.002260	0.004635	1.96	3.16
C. jacchus	0.001804	0.001450	0.000404	0.001444	0.002367	0.005257	1.28	0.41
C. torquatus	0.001807	0.001807	0.000080	0.001861	0.002302	0.004924	0.94	0.09
E. fulvus	0.002305	0.001778	0.000243	0.001770	0.003109	0.007291	0.49	0.43
G. gorilla	0.002642	0.002400	0.001158	0.002499	0.003223	0.006645	0.41	4.11
H. griseus	0.002209	0.001737	0.000467	0.001709	0.003143	0.006790	0.76	1.58
H. Lar	0.002684	0.002379	0.000409	0.002424	0.003640	0.007538	1.16	1.91
H. moloch	0.002626	0.002285	0.000214	0.002295	0.003685	0.007471	1.84	0.44
H. muelleri	0.002814	0.002393	0.000147	0.002448	0.003994	0.008316	1.11	2.33
H. sapiens	0.002600	0.002135	0.000038	0.002230	0.002706	0.005418	1.67	3.18
L. catta	0.002917	0.002446	0.000215	0.002463	0.003778	0.008997	0.70	0.71
M. fascicularis	0.001793	0.001727	0.000658	0.001749	0.002468	0.004300	0.47	1.25
M. fuscata	0.002072	0.001948	0.001142	0.001871	0.002633	0.004804	0.29	3.95
M. mulatta	0.002212	0.002235	0.000807	0.002184	0.002962	0.005278	0.56	2.26
M. nemestrina	0.001836	0.001649	0.000607	0.001717	0.002390	0.004504	0.46	4.11
N. coucang	0.001753	0.001519	0.000586	0.001538	0.002197	0.004426	0.03	1.19
P. cynocephalus	0.002195	0.002066	0.000712	0.002113	0.003057	0.005958	1.03	4.65
P. Pithecia	0.001601	0.001268	0.000637	0.001248	0.001989	0.004517	0.05	1.59
P. pygmaeus	0.002656	0.002245	0.000557	0.002353	0.003428	0.007235	1.76	4.82
P. troglodytes	0.002334	0.002165	0.000668	0.002265	0.002757	0.005647	0.78	2.28
P. ursinus	0.001973	0.001828	0.000323	0.001830	0.002856	0.005123	0.09	0.07
S. sciureus	0.001595	0.001378	0.000115	0.001405	0.002244	0.004310	1.00	1.99
T. cristatus	0.002222	0.002063	0.000499	0.002098	0.002960	0.005671	0.85	1.70
T. gelada	0.002528	0.002454	0.000696	0.002407	0.003564	0.006406	0.36	1.90

Table S7 Statistics from the Two-Way PERMANOVA for the four biting cases (IB: Incisive Bite, CB: Canine Bite, PB: Premolar Bite and MB: Molar Bite) when comparing diet and hardness in MWAM.

**IB: Incisive Bite**					
Source	Sum of sqrs	df	Mean square	F	p
DIET	3.65E-06	2	1.82E-06	6.1066	0.0041
HARDNESS	3.28E-06	1	3.28E-06	11.001	0.0019
Interaction	-1.36E-07	2	-6.82E-08	-0.22846	0.3993
Residual	7.46E-06	25	2.99E-07		
Total	1.43E-05	30			
**CB: Canine Bite**					
Source	Sum of sqrs	df	Mean square	F	p
DIET	3.48E-06	2	1.74E-06	6.809	0.0024
HARDNESS	3.58E-06	1	3.58E-06	14.03	0.0009
Interaction	1.78E-07	2	8.88E-08	0.34794	0.2789
Residual	6.38E-06	25	2.55E-07		
Total	1.36E-05	30			
**PB: Premolar Bite**					
Source	Sum of sqrs	df	Mean square	F	p
DIET	2.62E-06	2	1.31E-06	4.6505	0.0151
HARDNESS	3.63E-06	1	3.63E-06	12.902	0.0013
Interaction	-6.14E-07	2	-3.07E-07	-1.0903	0.6727
Residual	7.04E-06	25	2.82E-07		
Total	1.27E-05	30			
**MB: Molar Bite**					
Source	Sum of sqrs	df	Mean square	F	p
DIET	1.31E-06	2	6.56E-07	5.5914	0.0079
HARDNESS	1.34E-06	1	1.34E-06	11.451	0.0023
Interaction	-3.13E-07	2	-1.57E-07	-1.3356	0.8343
Residual	2.93E-06	25	1.17E-07		
Total	5.28E-06	30			

Table S8 Statistics from the Two-Way PERMANOVA for the four biting cases (IB: Incisive Bite, CB: Canine Bite, PB: Premolar Bite and MB: Molar Bite) when comparing diet and hardness in MWM.

**IB: Incisive Bite**					
Source	Sum of sqrs	df	Mean square	F	p
DIET	2.12E-06	2	1.06E-06	5.572	0.0082
HARDNESS	1.49E-06	1	1.49E-06	7.8441	0.0076
Interaction	1.42E-07	2	7.10E-08	0.37358	0.2112
Residual	4.75E-06	25	1.90E-07		
Total	8.51E-06	30			
**CB: Canine Bite**					
Source	Sum of sqrs	df	Mean square	F	p
DIET	2.15E-06	2	1.07E-06	8.9798	0.0005
HARDNESS	1.57E-06	1	1.57E-06	13.095	0.0007
Interaction	2.40E-07	2	1.20E-07	1.0031	0.1322
Residual	2.99E-06	25	1.20E-07		
Total	6.95E-06	30			
**PB: Premolar Bite**					
Source	Sum of sqrs	df	Mean square	F	p
DIET	1.51E-06	2	7.54E-07	5.2641	0.0084
HARDNESS	1.60E-06	1	1.60E-06	11.19	0.0022
Interaction	-2.48E-07	2	-1.24E-07	-0.86646	0.628
Residual	3.58E-06	25	1.43E-07		
Total	6.44E-06	30			
**MB: Molar Bite**					
Source	Sum of sqrs	df	Mean square	F	p
DIET	1.32E-06	2	6.62E-07	4.781	0.0125
HARDNESS	7.38E-07	1	7.38E-07	5.3255	0.0241
Interaction	-1.78E-07	2	-8.90E-08	-0.64271	0.5243
Residual	3.46E-06	25	1.39E-07		
Total	5.35E-06	30			

Table S9 Statistics from the Two-Way PERMANOVA for the four biting cases (IB: Incisive Bite, CB: Canine Bite, PB: Premolar Bite and MB: Molar Bite) when comparing diet and hardness in all the percentiles together M(25%), M(50%), M(75%) and M(95%)

**IB: Incisive Bite**					
Source	Sum of sqrs	df	Mean square	F	p
DIET	3.79E-05	2	1.89E-05	5.2196	0.0067
HARDNESS	4.37E-05	1	4.37E-05	12.058	0.0011
Interaction	4.51E-06	2	2.26E-06	0.62209	0.2046
Residual	9.07E-05	25	3.63E-06		
Total	1.77E-04	30			
**CB: Canine Bite**					
Source	Sum of sqrs	df	Mean square	F	p
DIET	3.49E-05	2	1.74E-05	4.6422	0.0116
HARDNESS	4.94E-05	1	4.94E-05	13.147	0.0008
Interaction	7.37E-06	2	3.69E-06	0.98191	0.1652
Residual	9.38E-05	25	3.75E-06		
Total	1.85E-04	30			
**PB: Premolar Bite**					
Source	Sum of sqrs	df	Mean square	F	p
DIET	3.20E-05	2	1.60E-05	4.162	0.0172
HARDNESS	4.35E-05	1	4.35E-05	11.296	0.0016
Interaction	2.86E-06	2	1.43E-06	0.37126	0.2797
Residual	9.62E-05	25	3.85E-06		
Total	1.75E-04	30			
**MB: Molar Bite**					
Source	Sum of sqrs	df	Mean square	F	p
DIET	2.10E-05	2	1.05E-05	8.1183	0.0006
HARDNESS	1.96E-05	1	1.96E-05	15.162	0.0005
Interaction	1.69E-06	2	8.47E-07	0.65454	0.157
Residual	3.23E-05	25	1.29E-06		
Total	7.46E-05	30			

Figure S1- Box-plots of the MWAM and M(95%) values of all analysed species grouped by hardness of ingesta (H: hard eaters; S: soft eaters) and by dietary categories (O: omnivore; F: frugivore and L: folivore). IB: incisive bite; CB: canine bite; PB: premolar bite and MB: molar bite. The median is the middle line of the box and whiskers represent the range.

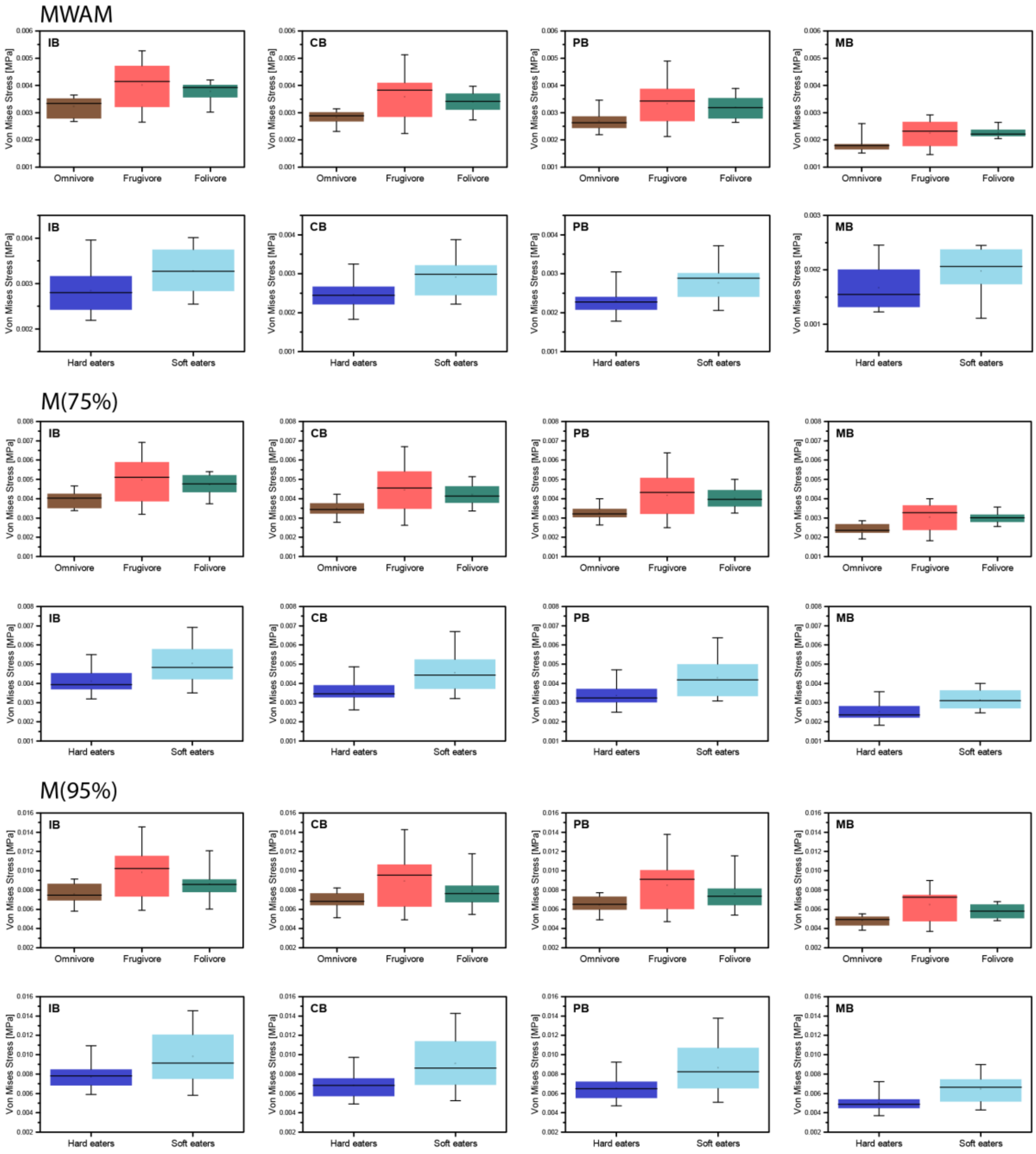

Figure S2 MWAM values for each species mapped on the phylogeny for the four biting scenarios. The values at nodes and branches were reconstructed using a maximum-likelihood ancestral character estimation method based on a Brownian motion model of evolution. The colour ranges from red representing higher average stress values, to blue, representing lower stress values.

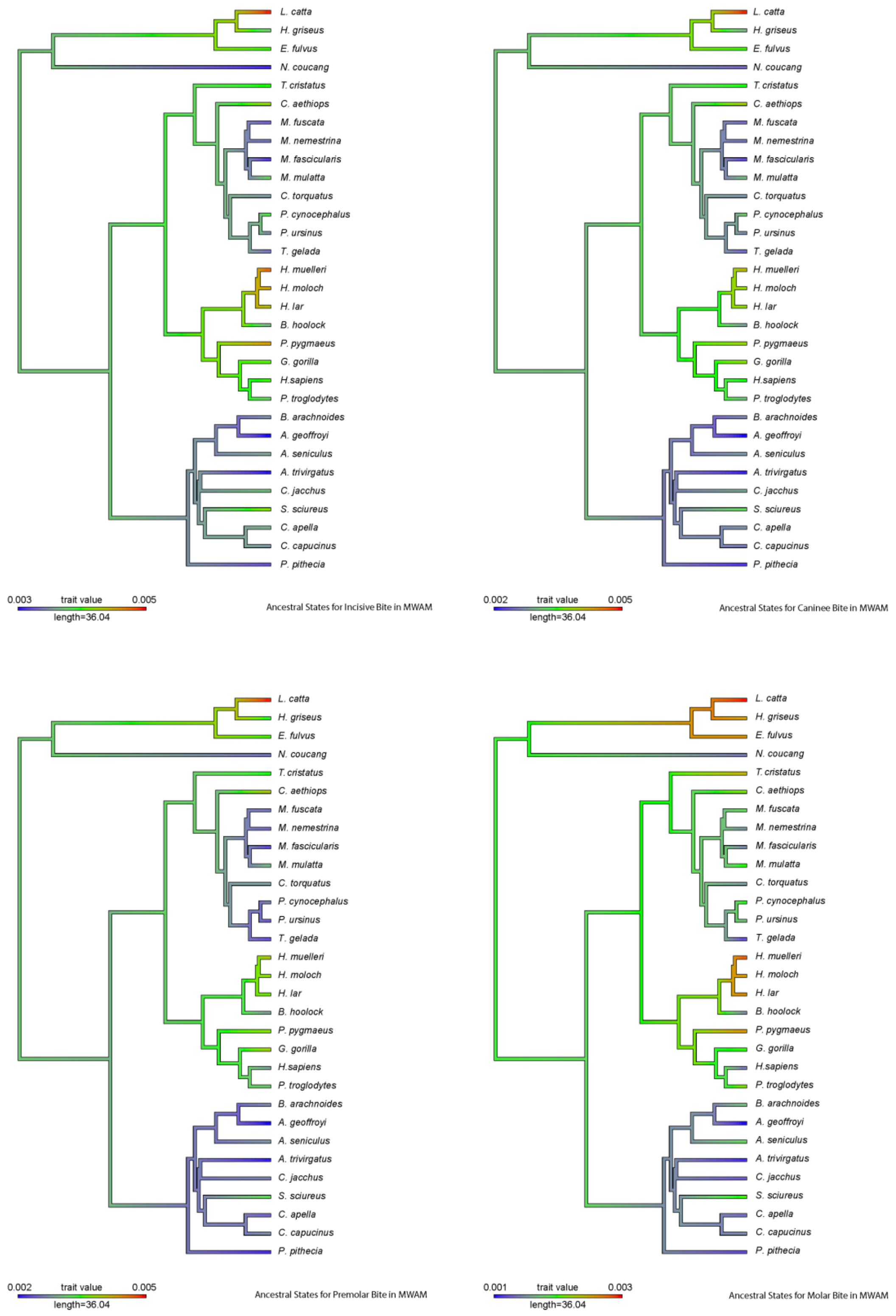

Figure S3 M(75%) values for each species mapped on the phylogeny for the four biting scenarios. The values at nodes and branches were reconstructed using a maximum-likelihood ancestral character estimation method based on a Brownian motion model of evolution. The colour ranges from red representing higher average stress values, to blue, representing lower stress values.

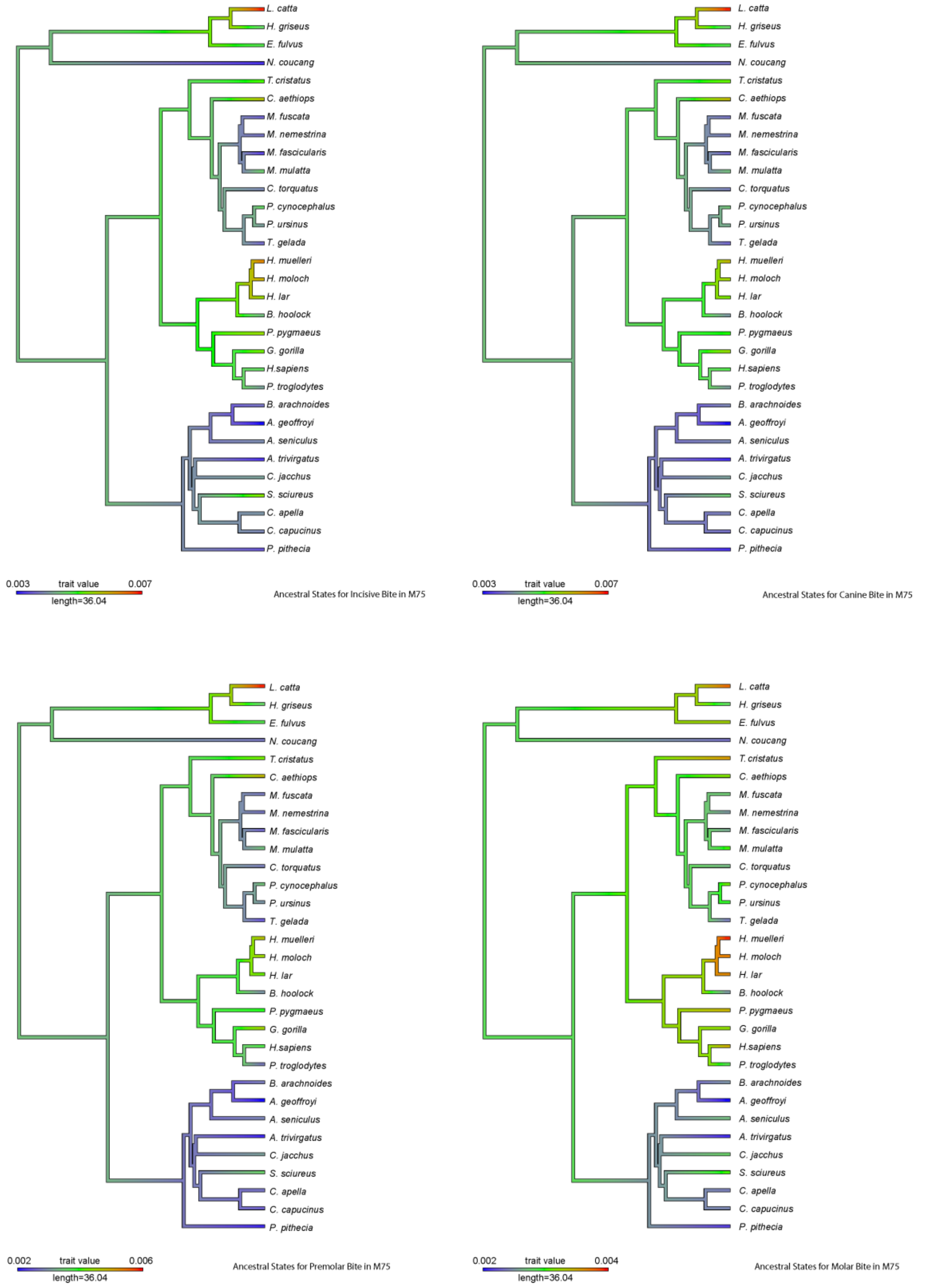

Figure S4 M(95%) values for each species mapped on the phylogeny for the four biting scenarios. The values at nodes and branches were reconstructed using a maximum-likelihood ancestral character estimation method based on a Brownian motion model of evolution. The colour ranges from red representing higher average stress values, to blue, representing lower stress values.

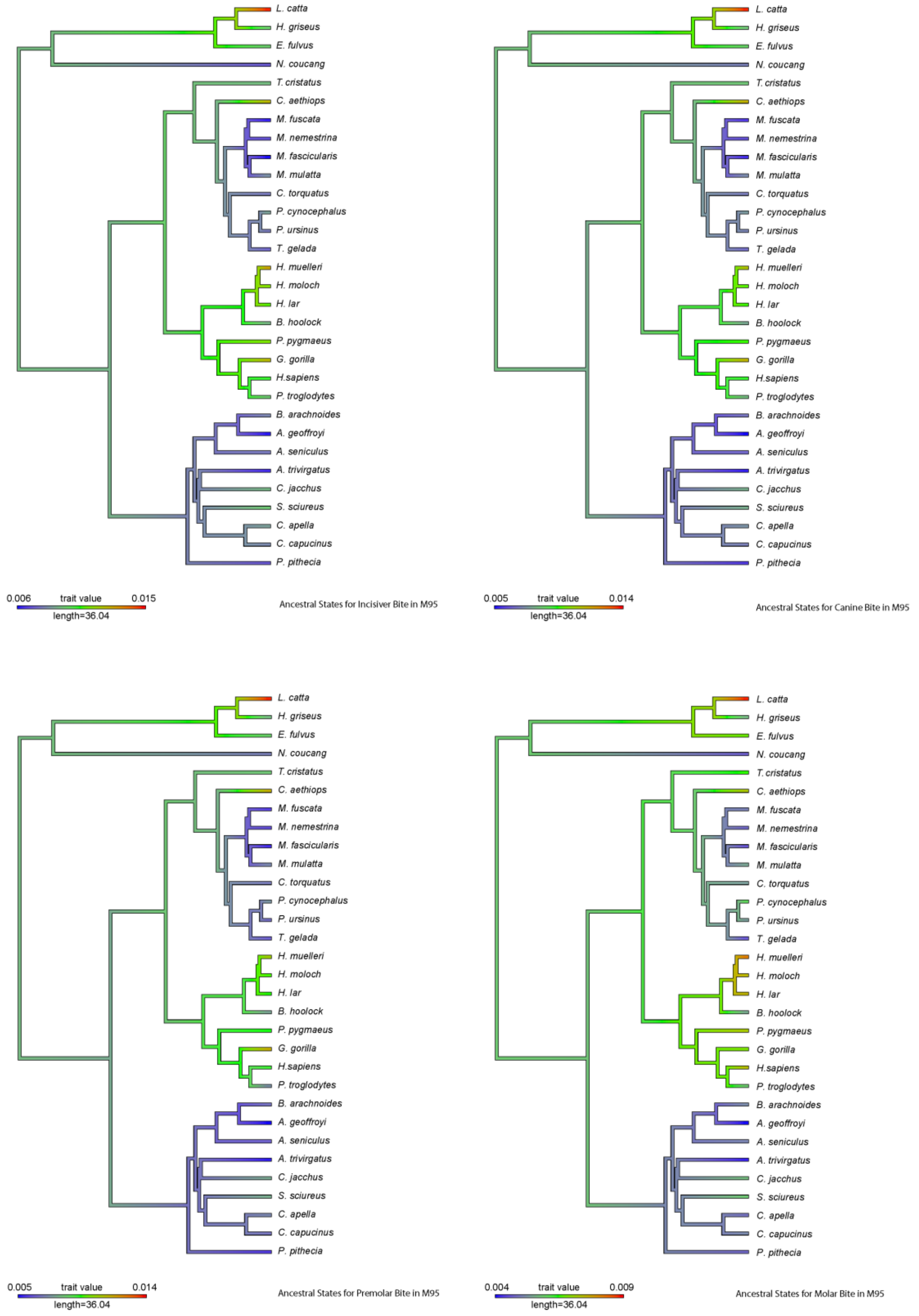

Supplement: Supplementary file 1 — Supplementary information [file 41598_2017_8161_MOESM1_ESM.pdf]
